# Supplementary material for: Cyclic AMP induces reversible EPAC1 condensates that regulate histone transcription
Source: Nat Commun. 2023 Sep 8;14:5521. doi: 10.1038/s41467-023-41088-x (PMC10491619; doi:10.1038/s41467-023-41088-x)
Supplement: Supplementary file 1 — Supplementary Information [file 41467_2023_41088_MOESM1_ESM.pdf]

Supplementary Information

Cyclic AMP induces reversible EPAC1 condensates that regulate histone transcription

Liliana Felicia Iannucci[1,2], Anna Maria D'Erchia[3], Ernesto Picardi[3], Daniela Bettio[4,5], Filippo Conca[1,2], Nicoletta Concetta Surdo[2,6], Giulietta Di Benedetto[2,6], Deborah Musso[1], Cristina Arrigoni[1], Marco Gaetano Lolicato[1], Mauro Vismara[1,2], Francesca Grisan[2], Leonardo Salviati[4,5], Luciano Milanesi[7], Graziano Pesole[3], and Konstantinos Lefkimmiatis[1,2,6,*].

1. Department of Molecular Medicine, University of Pavia, Pavia, Italy
2. Veneto Institute of Molecular Medicine, 35129 Padova, Italy
3. Department of Biosciences, Biotechnologies and Environment, University of Bari "Aldo Moro", Bari Italy
4. Clinical Genetics Unit, Department of Women and Children's Health, University of Padova
5. Fondazione Istituto di Ricerca Pediatrica Città della Speranza, Padova, Italy
6. Neuroscience institute, Italian National Research Council, Padova, Italy
7. Institute of Biomedical Technologies, Italian National Research Council, Milan, Italy

* Corresponding author: konstantinos.lefkimmiatis@unipv.it

Supplementary Figures 1-7

Supplementary Table 1

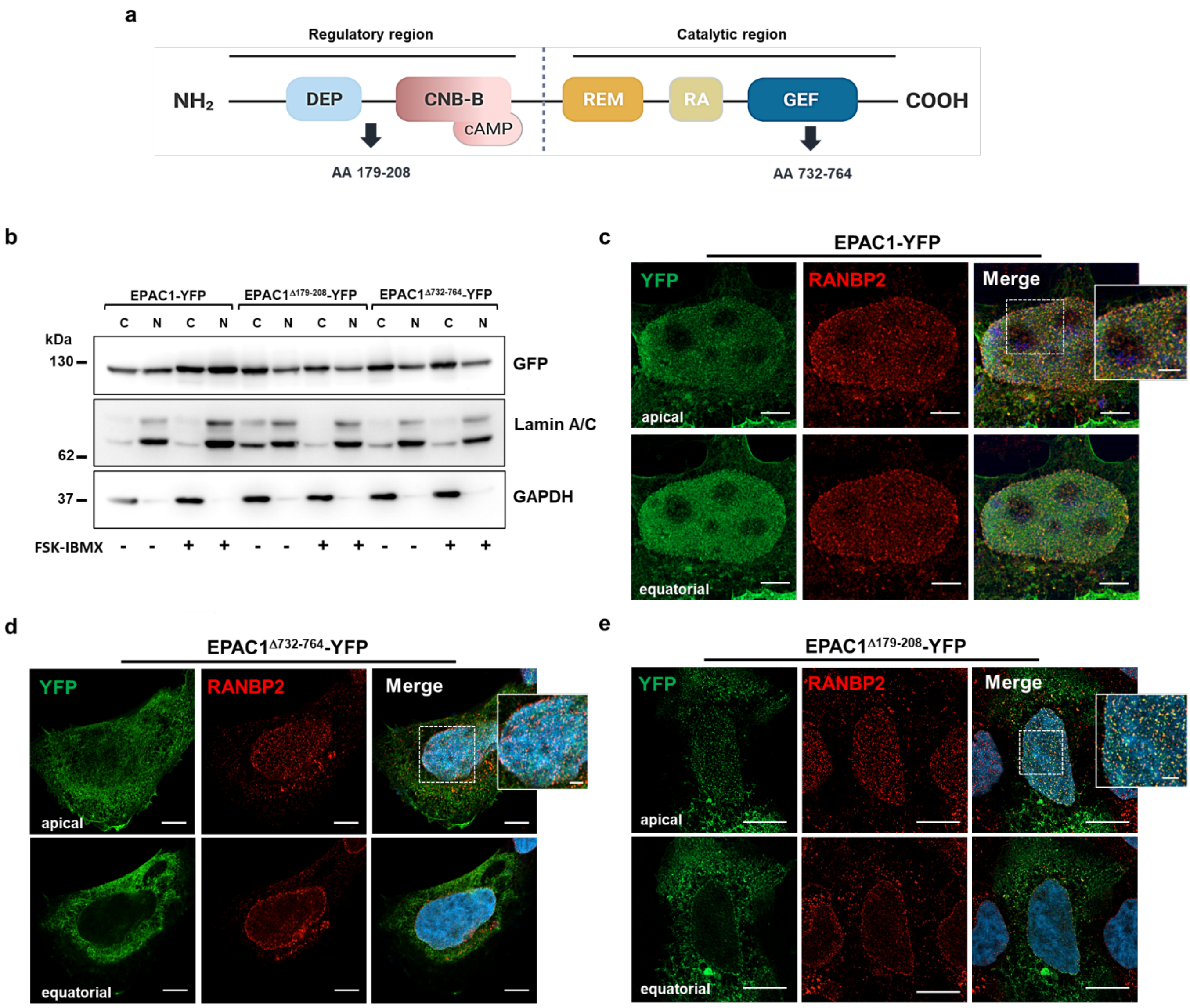

Supplementary Fig. 1| Two distinct amino acidic regions regulate the entry of EPAC1 in the nucleus. a Schematic representation of putative nuclear localization sequences within EPAC1 identified using the NLS Mapper algorithm. **b** Western blotting of cytosol and nuclei-enriched fractions of HEK cells expressing EPAC1-YFP, EPAC1$^{\Delta179\text{-}208}$-YFP and EPAC1$^{\Delta732\text{-}764}$-YFP. As compared to the WT construct, nuclear localization of both deletion mutants was hindered. Lamin A/C and GAPDH were used as nuclear and cytosolic markers respectively. Confocal images of cells expressing EPAC1-YFP (**c**), EPAC1$^{\Delta179\text{-}208}$-YFP (**d**) and EPAC1$^{\Delta732\text{-}764}$-YFP (**e**) probed for endogenous RANBP2 (red) (apical and equatorial views). Nuclei were labeled using DAPI (blue). C: cytosol; N: nucleus. Experiments were repeated at least two times with similar results. All scale bars, 10µm except enlargements (2µm).

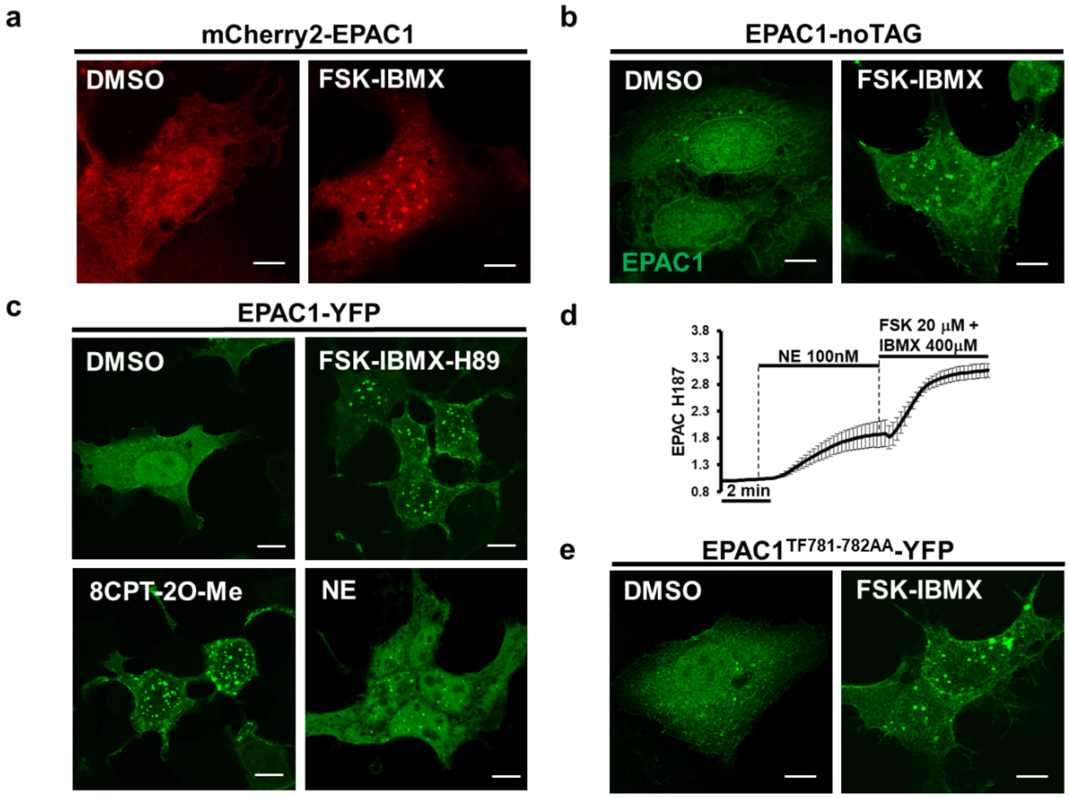

Supplementary Fig. 2| EPAC1 oligomerization is independent of PKA and EPAC1 activity.
a Confocal photomicrographs of HEK cells expressing an EPAC1 construct tagged with the monomeric fluorescent protein mCherry2 to its carboxy terminus. **b** Confocal photomicrographs of HEK cells expressing untagged EPAC1 and probed with a specific anti-EPAC1 antibody. **c** Confocal photomicrographs of HEK cells expressing EPAC1-YFP and treated with DMSO (vehicle), FSK-IBMX combined to the PKA inhibitor H89 (30μM), the EPAC specific cell permeable cAMP analog 8CPT-cAMP (5μM) or norepinephrine (NE), 100nM. **d** FRET-based experiment using the cAMP sensor EPAC^{H187} demonstrate that NE treatment induces roughly 50% of the cAMP production induced by FSK-IBMX. Data are presented as mean values ± SD, n=5 independent experiments. Source data are provided as a Source Data file. **e** Confocal images of the catalytically dead mutant EPAC1$^{TF781-782AA}$-YFP. Nuclei were stained using DAPI. [FSK] 20μM, [IBMX] 400μM. Scale bar, 10μm. Experiments were repeated at least three times with similar results.

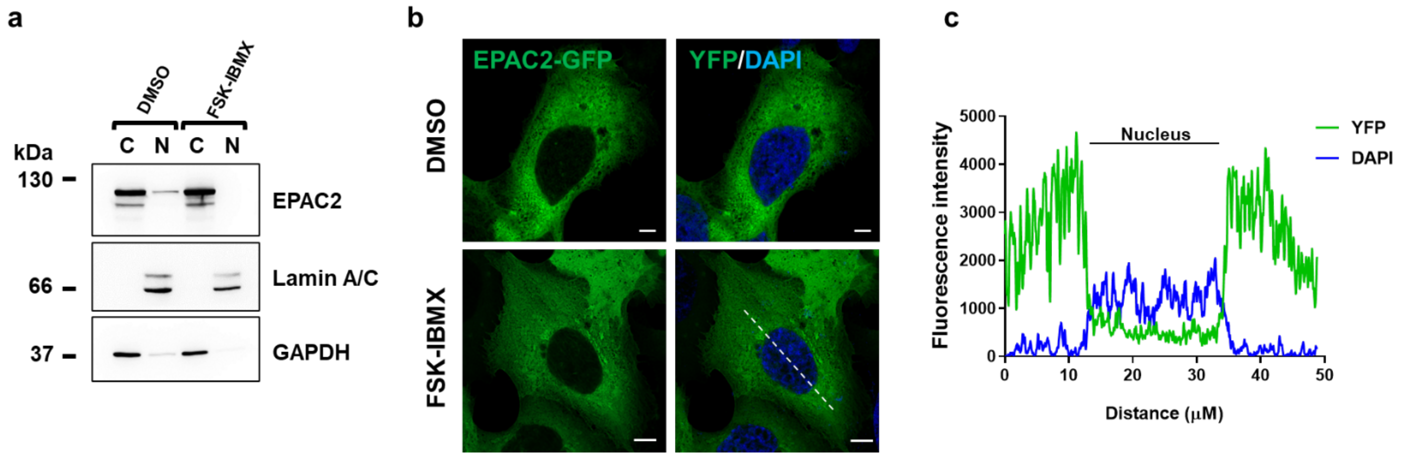

Supplementary Fig. 3| EPAC2 localizes in the cytosol of HEK cells and does not form biomolecular condensates in response of cAMP elevations.

a Western blotting of cytosol and nuclei-enriched fractions of HEK cells. Immunoblot of endogenous EPAC2 shows its primarily cytosolic localization. **b** Confocal photomicrographs of HEK cells expressing exogenous EPAC2-GFP treated with DMSO (control) or forskolin in combination to IBMX (FSK-IBMX) to increase cAMP levels. Nuclei were stained using DAPI (blue); all scale bars, 10μm. **c** Representative graph of fluorescence intensity across the cytosol and nuclei of an HEK cell expressing EPAC2-GFP. Experiments were repeated at least three times with similar results. Lamin A/C and GAPDH nuclear and cytosolic markers respectively. Nuclei were visualized using DAPI. C: cytosol; N: nucleus.

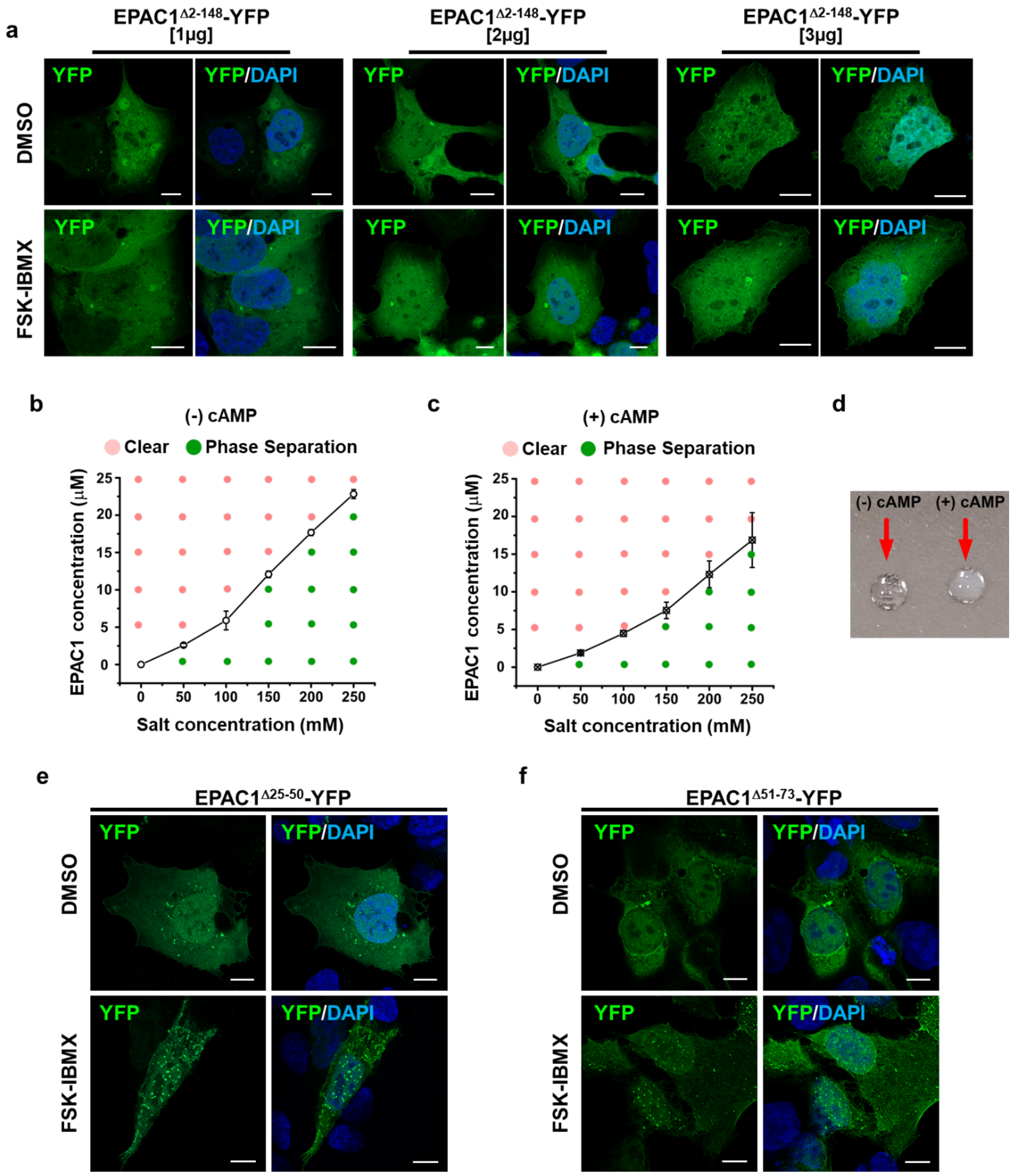

Supplementary Fig. 4| Specific residues of the N-terminal region of EPAC1 regulate its phase separation. **a** Confocal photomicrographs of HEK cells transfected with increasing doses of EPAC1$^{\Delta 2\text{-}148}$-YFP and treated with FSK/IBMX to increase cAMP levels or vehicle (DMSO). **b, c** Graphs representing the relation of purified recombinant EPAC1 concentration (Csat) in function of the concentration. Data are presented as mean values ± SEM. n=3 independent experiments. Source data are provided as a Source Data file. **d** Phase separation of 25 μM EPAC1 occurring *in vitro* at room temperature after the addition of 30 μM cAMP. **e**, **f** Confocal images of HEK cells expressing the deletion mutants EPAC1$^{\Delta 25\text{-}50}$-YFP or EPAC1$^{\Delta 51\text{-}73}$-YFP. Both mutants were able to form condensates in response to cAMP elevations induced by FSK-IBMX. Nuclei were stained using DAPI. [FSK] 20μM, [IBMX] 400μM. Scale bars, 10μm. Experiments were repeated at least three times with similar results.

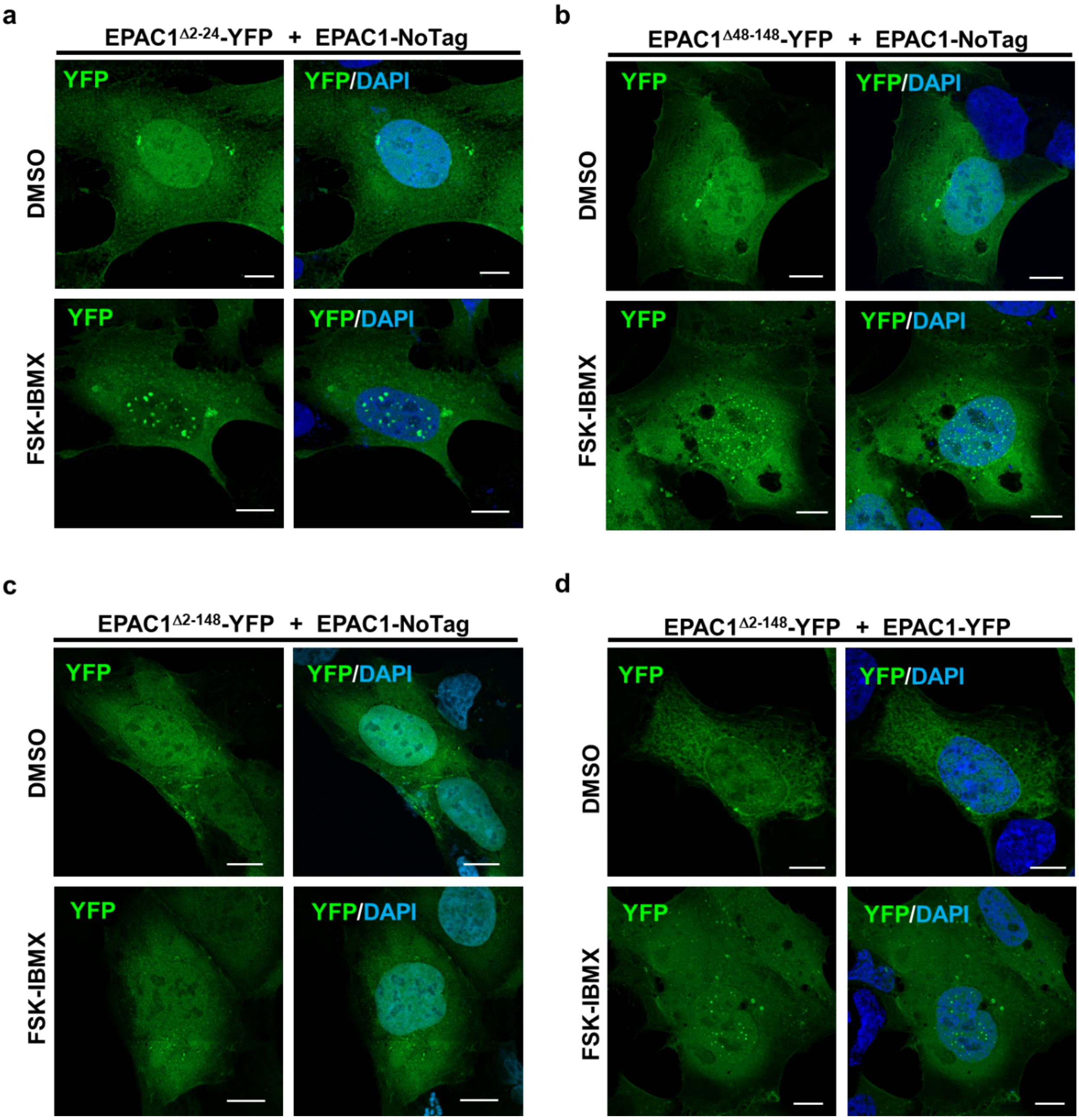

Supplementary Fig. 5| Mutants of the N-terminal region of EPAC1 are unable to phase separate independently but can participate in condensate formation.

Confocal photomicrographs of HEK cells expressing the condensation deficient mutants EPAC1$^{\Delta 2\text{-}24}$-YFP (**a**) or EPAC1$^{\Delta 48\text{-}148}$-YFP (**b**) together with an untagged EPAC1 wild type construct (EPAC1-no Tag). In response to increased cAMP levels (FSK-IBMX) YFP-labeled puncta appeared in the nuclei, indicating that both mutants, while unable to trigger independently puncta formation, were able to participate to the formation of condensates constituted by unlabeled wild type EPAC1. Confocal images of HEK cells expressing the condensation deficient mutants EPAC1$^{\Delta 2\text{-}148}$-YFP together with an untagged EPAC1 wild type (EPAC1-no Tag) (**c**) or a YFP tagged (EPAC1-YFP) construct (**d**). When co-expressed with the untagged construct, no YFP-labeled condensates appeared while EPAC1-YFP was able to give rise to condensates in the presence of EPAC1$^{\Delta 2\text{-}148}$-YFP indicating that the latter did not inhibit condensate formation. Scale bar, 10µm. Experiments were repeated for three times independently with similar results.

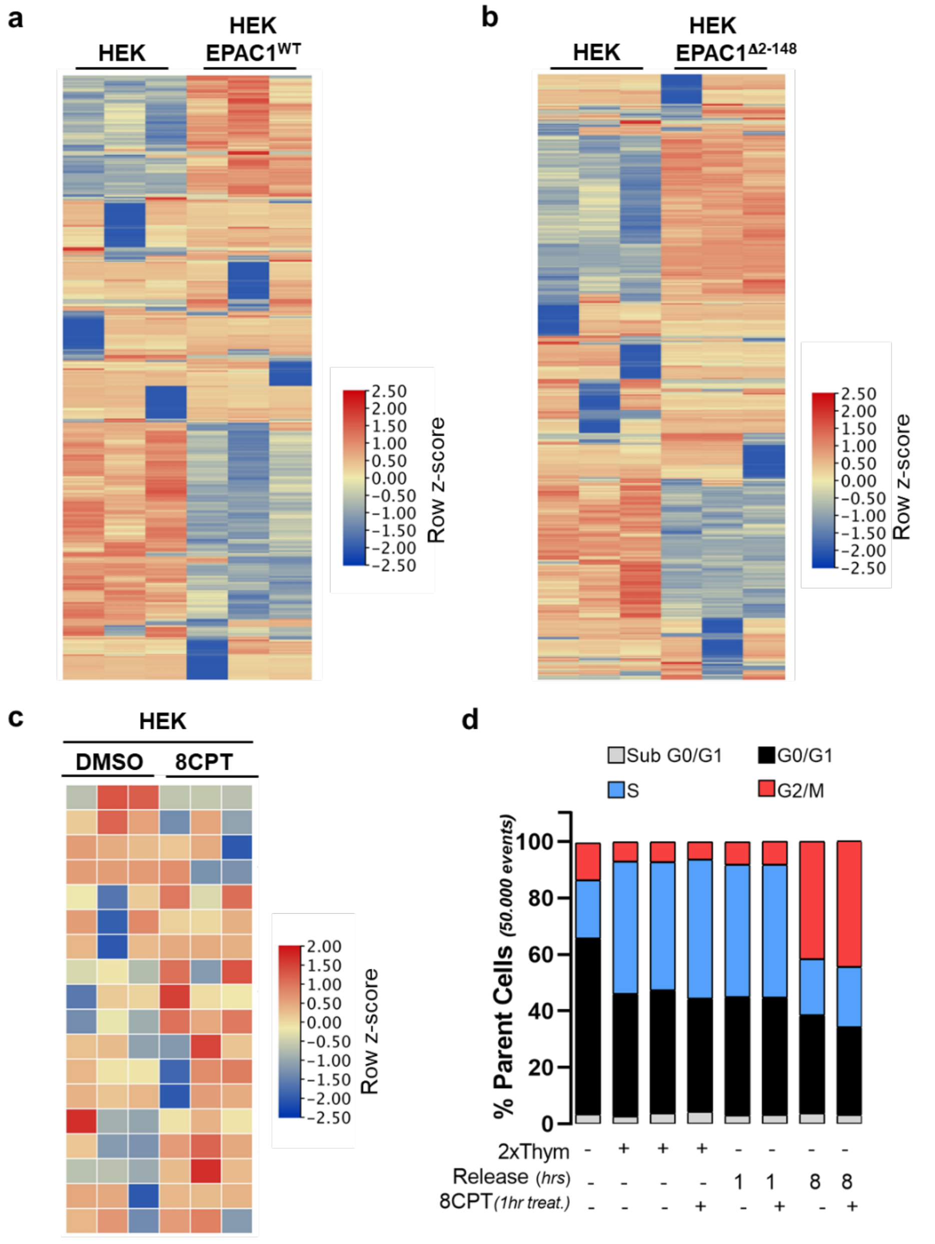

Supplementary Fig. 6| Overexpression of EPAC1-YFP or EPAC1$^{\Delta 2\text{-}148}$-YFP changes the transcriptional signatures of EPAC1-deficient HEK cells. Heatmap of gene expression comparing the expression signatures of naïve HEK cells to those of HEK cells expressing EPAC1-YFP (**a**) or EPAC1$^{\Delta 2\text{-}148}$-YFP (**b**). **c** Heatmap of naïve HEK cells treated with vehicle (DMSO) or the EPAC1-specific cell permeant cAMP analog 8CPT-cAMP (5μM). **d** Cell cycle assay in HEK cells synchronized in S phase with 2x Thymidine (Thym) block. Cells were released for 1 or 8 hours in the presence of DMSO (vehicle) or 8CPT-cAMP treatment of which nether affected the cell cycle progression at both timings from thymidine release. Experiments for **a**, **b**, **c** were repeated three times independently while experiments for **d** were repeated twice. Source data are provided as a Source Data file.

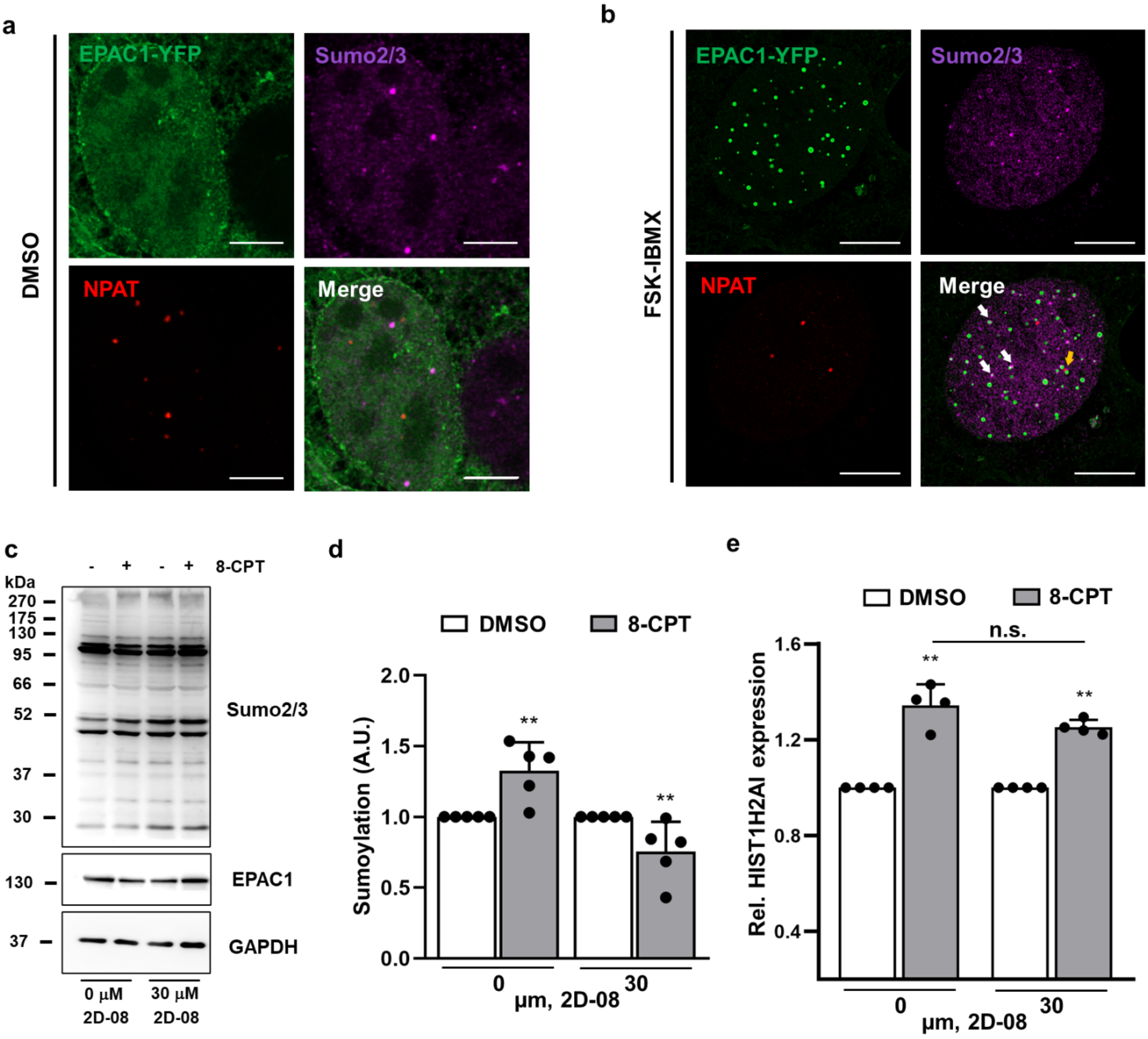

Supplementary Fig. 7|EPAC1 condensates affect histone transcription independently of SUMOylation. Confocal photomicrographs of HEK cells expressing EPAC1-YFP (green) and probed with specific antibodies for endogenous SUMO2/3 (magenta) and NPAT (red), treated with DMSO (**a**) or FSK-IBMX (**b**). White arrows indicate foci labeled with SUMO2/3 (magenta) and nEPAC1-YFP while yellow arrows indicates NPAT and EPAC1-YFP condensate interaction. Nuclei were stained using DAPI. [FSK] 20µM, [IBMX] 400µM. Scale bar 10µm. Experiments were repeated at least three times with similar results. **c** Western blotting of HEK cells expressing EPAC1-YFP treated with the SUMOylation inhibitor 2D-08 for 24 hours and challenged with DMSO or 8CPT-cAMP, [2D-08] 30µM, [8CPT-cAMP] 5µM. **d** Quantification of total SUMOylation in response to DMSO and 8CPT-cAMP in the presence or absence of 2D-08. [2D-08] 30µM, [8CPT-cAMP] 5 µM. Data from five independent experiments, statistical significance was determined by unpaired Kolmogorov–Smirnov test (**p= 0,0079) error bars ± SD. **e** Real-Time qPCR in HEK cells expressing EPAC1-YFP testing the expression of Hist1h2ai (representative of the expression of Chr 6p22.2 locus) treatment with 8CPT-cAMP consistently increased the expression of Hist1h2ai and this effect persisted independently of the presence of 2D-08 to inhibit SUMOylation. Data from four independent experiments, statistical significance was determined by two-sided, unpaired t-test with Welch's correction (**p= 0,0044; ***p=0,0005) error bars ± SD. Source data are provided as a Source Data file.

Supplementary Table 1: Sequences of the primers used for cloning and mutagenesis of EPAC1

Oligonucleotide Name	Oligonucleotide Sequence (5' to 3')
EPAC1_HindIII_C1Fw	CTCAAGCTTCCATGGTGTTGAGAAGGATGCA
EPAC1_KpnI_C1Rev	CGCGGTACCTCATGGCTCCAGCTCTCGGGAG
Epac1_D2-24_Fw	CTAGCATGTGCATCCAGGGGCTGCGC
Epac1_D2-24_Rev	GGATGCACATGCTAGCGGATCTGACGGTTC
Ep_Del25-50_Fw	GTCCGAGCTCCACAGAGCGGGTGCTC
Ep_Del25-50_Rev	CTGTGGAGCTCGGACGCTGGTGCTC
Epac1_Del48-148_Fw	AGAGCCTGCCCGTGGGAACTCATGAGATGG
Epac1_Del48-148_Rev	CCACGGGCAGGCTCTCGCTGAAATCCAG
Ep_Del51-73_Fw	AGCAGGCCATCCGAGACCGGAAGTACCACC
Ep_Del51-73_Rev	AGCAGGCCATCCGAGACCGGAAGTACCACC
Ep_Del179-208_Fw	CTGTGGCAAACTCGGTGAAGCGAGAATTAGCG
Ep_Del179-208_Rev	CCGAGTTTGCCACAGTGAGCAGGGC
Ep_Del732-764_Fw	ACACCTGGCTCTCCCCTCCTGTCATCCCC
Ep_Del732-764_Rev	GGGAGAGCCAGGTGTGGGCTAGGCG
Ep1_D145-175_Fw	TCCCCGGGACTGTGGCACTTCGAAAGCC
Ep1_ D145-175_Rev	CCACAGTCCCGGGGAACCGGTAGAATTGG
Ep1_TF781-782AA_Fw	ACATGGCCGCCATTCATGAGGGAAACCACACACT
Ep1_ TF781-782AA_Rev	GAATGGCGGCCATGTCTTTGAGAAGAAGGGGCATG
