# Supplementary material for: Cyclic AMP induces reversible EPAC1 condensates that regulate histone transcription
Source: Nat Commun. 2023 Sep 8;14:5521. doi: 10.1038/s41467-023-41088-x (PMC10491619; doi:10.1038/s41467-023-41088-x)
Supplement: Supplementary file 3 — Description of Additional Supplementary Files [file 41467_2023_41088_MOESM3_ESM.pdf]

Cyclic AMP-induced reversible EPAC1 condensates regulate histone transcription

Liliana Felicia Iannucci, Anna Maria D'Erchia, Ernesto Picardi, Daniela Bettio, Filippo Conca, Nicoletta Concetta Surdo, Giulietta Di Benedetto, Deborah Musso, Cristina Arrigoni, Marco Lolicato, Mauro Vismara, Francesca Grisan, Leonardo Salviati, Luciano Milanesi, Graziano Pesole, and Konstantinos Lefkimmiatis.

Supplementary movie 1| EPAC1 nuclear puncta formation in response to cAMP elevation

Confocal live cell imaging of HEK293 cells transfected with EPAC1-YFP and challenged with FSK-IBMX. EPAC1 localizes in both cytosol and nucleus. After 5 minutes of treatment cytosolic EPAC1 moves to the plasma membrane while nuclear EPAC1 forms round structures that disassemble upon rinsing of the stimulus.

Supplementary movie 2| EPAC1 forms reversible nuclear puncta.

Confocal live cell imaging of HEK293 cells transfected with EPAC1-YFP and pre-treated with FSK-IBMX to induce nuclear EPAC1 condensates formation. Upon rinsing the stimuli nuclear EPAC1 puncta rapidly disassembled and reformed in the subsequent round of stimulation with FSK-IBMX.

Supplementary movie 3| 3D rendering of EPAC1 condensates.

Representative 3D rendering of two nuclear EPAC1 condensates acquired in super-resolution confocal microscopy.

Supplementary movie 4| 1,6 Hexanediol effect on EPAC1 condensates.

Confocal live cell imaging of HEK293 cells expressing EPAC1-YFP and pre-treated with FSK-IBMX to induce nuclear EPAC1 condensates formation. Cells were then challenged with 5% 1,6 Hexanediol which alters weak hydrophobic interaction leading to rapid disassemble of nuclear EPAC1 condensates.

Supplementary movie 5| EPAC1 puncta fusion events.

Confocal live cell imaging of HEK293 cells expressing EPAC1-YFP and pre-treated with FSK-IBMX to induce nuclear EPAC1 condensates formation. Arrows indicate two fusion events between adjacent condensates.

Supplementary movie 6| Representative FRAP experiment of EPAC1 condensates.

Confocal live cell imaging of HEK293 cells expressing EPAC1-YFP and pre-treated with FSK-IBMX to induce nuclear EPAC1 condensates formation. The movie shows a representative bleaching event and recovery (white arrow) of a single EPAC1 condensates in a FRAP experiment.
