# Supplementary material for: Cyclic AMP induces reversible EPAC1 condensates that regulate histone transcription
Source: Nat Commun. 2023 Sep 8;14:5521. doi: 10.1038/s41467-023-41088-x (PMC10491619; doi:10.1038/s41467-023-41088-x)
Supplement: Supplementary file 10 — Reporting Summary [file 41467_2023_41088_MOESM10_ESM.pdf]

nature portfolio

Corresponding author(s): Konstantinos Lefkimmiatis

Last updated by author(s): Aug 5, 2023

Reporting Summary

Nature Portfolio wishes to improve the reproducibility of the work that we publish. This form provides structure for consistency and transparency in reporting. For further information on Nature Portfolio policies, see our Editorial Policies and the Editorial Policy Checklist.

Statistics

For all statistical analyses, confirm that the following items are present in the figure legend, table legend, main text, or Methods section.

n/a	Confirmed	
☐	☒	The exact sample size (*n*) for each experimental group/condition, given as a discrete number and unit of measurement
☐	☒	A statement on whether measurements were taken from distinct samples or whether the same sample was measured repeatedly
☐	☒	The statistical test(s) used AND whether they are one- or two-sided<br>*Only common tests should be described solely by name; describe more complex techniques in the Methods section.*
☒	☐	A description of all covariates tested
☒	☐	A description of any assumptions or corrections, such as tests of normality and adjustment for multiple comparisons
☐	☒	A full description of the statistical parameters including central tendency (e.g. means) or other basic estimates (e.g. regression coefficient) AND variation (e.g. standard deviation) or associated estimates of uncertainty (e.g. confidence intervals)
☐	☒	For null hypothesis testing, the test statistic (e.g. *F*, *t*, *r*) with confidence intervals, effect sizes, degrees of freedom and *P* value noted<br>*Give P values as exact values whenever suitable.*
☒	☐	For Bayesian analysis, information on the choice of priors and Markov chain Monte Carlo settings
☒	☐	For hierarchical and complex designs, identification of the appropriate level for tests and full reporting of outcomes
☒	☐	Estimates of effect sizes (e.g. Cohen's *d*, Pearson's *r*), indicating how they were calculated

Our web collection on statistics for biologists contains articles on many of the points above.

Software and code

Policy information about availability of computer code

Data collection	High resolution and confocal images were collected using a ZEISS LSM90O microscope equipped with ZEN blue 3.0 software;<br>FRET- imaging raw data were collected using a modified Cell-R software (version 2.2);<br>FRAP images were collected using a Leica SP5 Confocal microscope equipped with LasAF 2.6.0 software with the FRAP-Wizard module;<br>Flow cytometry was performed using FACSCanto II system equipped with BD FacsDiva 8.0 software;<br>Western blot images were collected using ImageQuant Las 4000 mini 1.2 software.
Data analysis	Fiji ImageJ 1.53c;<br>GraphPad prism 8.0.2;<br>Microsoft Excel 365.

For manuscripts utilizing custom algorithms or software that are central to the research but not yet described in published literature, software must be made available to editors and reviewers. We strongly encourage code deposition in a community repository (e.g. GitHub). See the Nature Portfolio guidelines for submitting code & software for further information.

Data

Policy information about availability of data

All manuscripts must include a data availability statement. This statement should provide the following information, where applicable:

- Accession codes, unique identifiers, or web links for publicly available datasets
- A description of any restrictions on data availability
- For clinical datasets or third party data, please ensure that the statement adheres to our policy

RNAseq data have been deposited in the public archive of high throughput sequencing data SRA (Sequence Read Archive) under accession number PRJNA1001149 [https://www.ncbi.nlm.nih.gov/bioproject/PRJNA1001149]. All uncropped gels and numerical values are provided in the Source data file. The accession number of the Human reference genome used in this study is GRCh38.p13. Source data are provided as a Source Data file. Source data are provided with this paper.

Research involving human participants, their data, or biological material

Policy information about studies with human participants or human data. See also policy information about sex, gender (identity/presentation), and sexual orientation and race, ethnicity and racism.

Reporting on sex and gender	NA
Reporting on race, ethnicity, or other socially relevant groupings	NA
Population characteristics	NA
Recruitment	NA
Ethics oversight	NA

Note that full information on the approval of the study protocol must also be provided in the manuscript.

Field-specific reporting

Please select the one below that is the best fit for your research. If you are not sure, read the appropriate sections before making your selection.

☒ Life sciences ☐ Behavioural & social sciences ☐ Ecological, evolutionary & environmental sciences

For a reference copy of the document with all sections, see nature.com/documents/nr-reporting-summary-flat.pdf

Life sciences study design

All studies must disclose on these points even when the disclosure is negative.

Sample size	The sample size for all experiments in this study was determined based on ad hoc considerations depending on the goal of each experiment and overall was based on the following considerations:<br>- Based on pilot experiments we confirmed that the variability within our experimental groups was low, thanks to both the robust phenotype and the experimental models (immortalized cell lines). To further decrease the effects of transfection efficiency on variability, we enriched the transfected cell population by FACS shorting.<br>- The probability of type I (false negative) and type II (false positive) errors was minimal thanks to the inducible nature of EPAC1 condensates and the generation of mutants unable to form condensates.<br>- The "smallest effect of difference" for these experiments was very robust as the observed phenotype (EPAC1 condensate formation) is an "all or none" effect.<br>Based on these considerations, our experience and widely published literature, we concluded that three independent biological replicates represent the minimal sample size for reaching statistical significance and robust reproducible conclusions. RNA seq experiments were performed in triplicate and a high number of reads (100 million) in order to ensure high accuracy of the hits.
Data exclusions	Data exclusion depended on the outcomes of the positive and negative controls used in each experiment. Only experiments where the controls failed were excluded form analysis.
Replication	The main experiments of this work were reproduced 5 or more times with reproducible results. Western Blotting experiments testing the presence of target proteins were repeated 3 times except some cases that were repeated 2 times. All experiments were reproducible in different cell types and cell batches.
Randomization	The use of randomization aims to prevent bias. In our experiments, treatment bias was mitigated by using different types of treatments and cells and always comparing the effects/phenotypes of EPAC1 wild type to its mutant versions. Human bias was mitigated by performing blinded experiments and also by different authors repeating the crucial experiments of the study.

Blinding	When possible, immunofluorescence experiments were blinded. Collection of samples for Western Blotting and RNAseq experiments was not blinded as the order of loading is crucial for the interpretation and analysis of these experiments.

Reporting for specific materials, systems and methods

We require information from authors about some types of materials, experimental systems and methods used in many studies. Here, indicate whether each material, system or method listed is relevant to your study. If you are not sure if a list item applies to your research, read the appropriate section before selecting a response.

Materials & experimental systems

n/a	Involved in the study
☐	☒ Antibodies
☐	☒ Eukaryotic cell lines
☒	☐ Palaeontology and archaeology
☒	☐ Animals and other organisms
☒	☐ Clinical data
☒	☐ Dual use research of concern
☒	☐ Plants

Methods

n/a	Involved in the study
☒	☐ ChIP-seq
☒	☐ Flow cytometry
☒	☐ MRI-based neuroimaging

Antibodies

Antibodies used

Primary antibodies: anti-GAPDH (H12) (Santa Cruz Biotechnology, sc-166574); anti-Lamin A/C (4C11) (Sigma, SAB4200236); anti-EPAC1 (5D3) (Cell Signalling Technologies, 4155); anti-EPAC2 (D3P3J) (Cell Signalling Technologies, 43239); anti-Sumo2/3 polyclonal (Enzo Life Sciences, BML-PW9465); anti-GFP polyclonal (Thermo Fisher Scientific, A11122); anti-βActin (AC15) (Abcam, 49900).
Secondary antibodies: Peroxidase (HRP) Anti-Rabbit IgG (H+L) Goat Secondary Antibody (Jackson ImmunoResearch, 111-035-003); AffiniPure Peroxidase (HRP) Anti-Mouse IgG (H+L) Goat Secondary Antibody (Jackson ImmunoResearch, 115-035-003).
Antibodies used for immunofluorescence
Primary antibodies: SMN (F-5) (Santa Cruz Biotechnology, sc-365909); Nucleolin polyclonal (Sigma-Aldrich, N2662); NPAT (27) (Santa Cruz Biotechnology, sc-136007); PML (Sigma-Aldrich, PLA-0172); EPAC1-488 (EPR1672) (Abcam, ab201506); Sumo 2/3 polyclonal (Abcam, ab3742); RanBP2 (D-4) (Santa Cruz Biotechnology, sc-74518).

Secondary antibodies: Goat anti Rabbit IgG (H+L) Secondary Antibody, Alexa Fluor 568 (Invitrogen, A-11011); Goat anti-Rabbit IgG (H+L) Cross-Adsorbed Secondary Antibody, Alexa Fluor 647 (Thermo Scientific, A-21244); Goat anti-Mouse IgG (H+L) Highly Cross-Adsorbed Secondary Antibody, Alexa Fluor Plus 647 (Thermo Scientific, A32728)

Validation

The primary Antibodies were chosen according to the websites citation. GAPDH and LaminA/C are the most common markers for nuclei/cytosol fractionation studies. Nuclear bodies' markers as well (SMN, nucleolin, PML and NPAT) were chosen according to citations and literature. Anti-SUMO2/3 (Enzo Life Sciences Inc., catalog no. MBL-PW9465) was recently used to investigate the relation of sumoylation and EPAC1 condensates.
EPAC1-488 validation was performed by visualizing overexpressed EPAC1-mcherry in HEK293. Using confocal microscopy we confirmed the overlap of the green fluorescence (EPAC1-488) to the red signals (EPAC1-mCherry), suggesting that this antibody specifically recognizes EPAC1. Similar experiments were performed by over expressing EPAC1 without a tag.

Eukaryotic cell lines

Policy information about cell lines and Sex and Gender in Research

Cell line source(s)	HEK293 cells were purchased from ATCC (ATCC number:CRL-1573)<br>Ovarian adenocarcinoma SK-OV-3 cells were purchased from ATCC (ATCC number: HTB-77)<br>Human Umbilical Vein Endothelial Cells (HUVEC) (ATCC, PCS-100-010) were a gift from the laboratory of Prof. Luca Scorrano (University of Padua).
Authentication	None of the cell lines used were authenticated
Mycoplasma contamination	All cell lines tested negative for mycoplasma contamination in recurrent tests every 3 months. In case of positive tests cell lines were eliminated and preventive treatment with azithromycin for a period of two weeks followed by a period of 2 weeks without the antibiotic and tested again to guarantee negativity.
Commonly misidentified lines<br>(See ICLAC register)	No common misidentified cell lines were used in this study.
